# Intrarenal Complement System Transcripts in Chronic Antibody-Mediated Rejection and Recurrent IgA Nephropathy in Kidney Transplantation

**DOI:** 10.3389/fimmu.2018.02310

**Published:** 2018-10-09

**Authors:** Marek Cernoch, Petra Hruba, Marek Kollar, Petra Mrazova, Lucia Stranavova, Alena Lodererova, Eva Honsova, Ondrej Viklicky

**Affiliations:** ^1^Transplant Laboratory, Transplant Center, Institute for Clinical and Experimental Medicine, Prague, Czechia; ^2^Department of Clinical and Transplant Pathology, Institute for Clinical and Experimental Medicine, Prague, Czechia; ^3^Department of Nephrology, Transplant Center, Institute for Clinical and Experimental Medicine, Prague, Czechia

**Keywords:** kidney transplantation, complement, chronic antibody-mediated rejection, IgA nephropathy, gene expression

## Abstract

**Background:** The complement system activation and regulation have been linked to post-transplant pathologies including chronic antibody mediated rejection (cAMR) and the recurrence of IgA nephropathy (ReIgAN) but distinct mechanisms remain to be elucidated.

**Methods:** In this retrospective single center study, the outcome of kidney transplantation was studied in 150 patients with late histological diagnosis to be either cAMR or ReIgAN, 14 stable kidney grafts at 3 months and finally 11 patients with native kidney IgAN nephropathy. To study a role of complement cascade and regulation in cAMR and ReIgAN, the RNA was extracted from available frozen kidney biopsy samples and using RT-qPCR transcripts of 11 target genes along with clinical data were determined and compared with stable grafts at 3 months protocol biopsies or IgAN native kidney nephropathy. Immunohistologically, CD46 (MCP), and C5 proteins were stained in biopsies.

**Results:** Interestingly, there were no differences in kidney graft survival between cAMR and ReIgAN since transplantation. cAMR was associated with significantly higher intragraft transcripts of *C3, CD59*, and *C1-INH* as compared to ReIgAN (*p* < 0.05). When compared to normal stable grafts, cAMR grafts exhibited higher *C3, CD55, CD59, CFH, CFI*, and *C1-INH* (*p* < 0.01). Moreover, ReIgAN was associated with the increase of *CD46, CD55, CD59* (*p* < 0.01), and *CFI* (*p* < 0.05) transcripts compared with native kidney IgAN. Rapid progression of cAMR (failure at 2 years after biopsy) was observed in patients with lower intrarenal CD55 expression (AUC 0.77, 78.6% sensitivity, and 72.7 specificity). There was highly significant association of several complement intrarenal transcripts and the degree of CKD regardless the diagnosis; *C3, CD55, CFH, CFI*, and *C1-INH* expressions positively correlated with eGFR (for all *p* < 0.001). Neither the low mRNA transcripts nor the high mRNA transcripts biopsies were associated with distinct trend in MCP or C5 proteins staining.

**Conclusions:** The intrarenal complement system transcripts are upregulated in progressively deteriorated kidney allografts.

## Introduction

Despite the advances in both the surgical procedures and immunosuppressive protocols, the long-term outcome of kidney transplantation has not substantially changed. The chronic antibody-mediated rejection is considered to be a major cause of late renal allograft failure (1–4). Besides chronic antibody-mediated rejection, the recurrence of glomerulonephritis affects substantial proportion of cases and decreases the graft survival as well (5). IgA nephropathy is the most frequent glomerular disease that frequently recurs after kidney transplantation (6).

The complement system involvement was shown to play a role in both chronic antibody-mediated rejection and IgA nephropathy pathogenesis (7–11). Both complement and complement regulators have been studied in relation to antibody-mediated rejection, and therapies based both on C5 blockade (12, 13) and C1 inhibition using the C1-inhibitor (14) or anti-C1 antibody (15) have been considered. However, the effect of complement activation and the significance of different components of the complement cascade are not fully understood when considering their impact on the long-term survival of the renal allograft. In this study, we evaluated several gene transcripts and proteins involved in complement regulation in main pathologies influencing the long-term kidney graft outcome, the chronic antibody mediated rejection (cAMR) and recurrent IgA nephropathy (ReIgAN) and showed that intrarenal complement transcripts are upregulated in progressively deteriorating kidney allografts regardless the underlying pathology.

## Materials and methods

### Patient's characteristics

This single center retrospective observational study was performed on a total cohort of 150 patients who had undergone kidney transplantation between 1988 and 2013; 93 patients (median age 49 years) with the biopsy-proven cAMR and 57 patients (median age 42 years) with the biopsy-proven ReIgAN were included. All graft biopsies (>12 months after transplantation) were performed between 2007 and 2015. The study protocol was approved by the Ethics Committee (No. A 13-02-01). Demographic and clinical characteristics are shown in Table 1. There was a statistically significant difference between both groups in creatinine levels at biopsy. Similarly, significantly higher maximum PRA levels and donor age were present in cAMR group. The cAMR group consisted from a higher proportion of retransplantations (27/93; 29%) compared to ReIgAN group (3/57; 5.3%).

**Table 1:** Demographic and clinical characteristics for the whole set of patients included in the study.

	**Chronic antibody-mediated rejection**			**Recurrent IgA nephropathy**			
Number of patients included	93			57			
Recipient gender (male/female)	57/36			46/11			*P* = 0.013
Age of recipient (years)	49 [9; 70]			42 [18; 68]			*P* = 0.052
Donor gender (male/female)	49/38			26/31			*P* = 0.208
Age of donor (years)	51.5 [16; 77]			47 [7;70]			*P* = 0.038
Donor type (cadaveric/living)	80/13			39/18			*P* = 0.010
Retransplantation (first/second/later)	66/22/5			54/3/-			
PRA max (%)	11 [0; 100]			4 [0; 87]			*P* = 0.007
HLA mismatch	4 [0; 6]			3 [0; 6]			*P* = 0.196
Cold ischemia (hours)	15.55 [0.45; 26.53]			14.24 [0.35; 24.68]			*P* = 0.359
Creatinine at the biopsy (μmol/L)	224.1 [98.0; 456.8]			197.1 [104.7; 616.2]			*P* = 0.014
Proteinuria at the biopsy (g/24 h)	2.14 [0.05; 11.58]			2.50 [0.13; 11.44]			*P* = 0.522
Dialysis vintage (years)	1.4 [0; 9.5]			1.5 [0; 7]			*P* = 0.453
Biopsy after transplantation (years)	6.03 [1; 20.65]			6.81 [1; 18.45]			*P* = 0.493
Immunosuppression prior to biopsy	Tacrolimus-based	Cyclosporin-based	Other	Tacrolimus-based	Cyclosporin-based	Other	
	73	14	6	31	22	4	*P* = 0.004

For the purpose of complement gene transcripts analyses we assembled an expression cohort of 51 kidney transplant recipients chosen from the total cohort, with the selection based on the availability of frozen tissue samples. Only the patients with samples sufficient for isolating the amount of RNA required for further analysis were included. For this part of the study, 26 patients (median age 46.5 years) with the biopsy-proven chronic antibody-mediated rejection and 25 patients (median age 39 years) with the biopsy-proven recurrence of IgA nephropathy (ReIgAN) were included. Demographic and clinical characteristics are shown in Table 2. The statistical analysis showed the groups to differ significantly in creatinine levels at the time of biopsy. Biopsies from 14 patients with normal histology and stable graft function at 3 month protocol biopsies and biopsies from 11 patients with proven native kidney IgAN were enrolled as controls (Table 3).

**Table 2:** Demographic and clinical characteristics for the set of patients selected for gene expression analysis.

	**Chronic antibody-mediated rejection**			**Recurrent IgA nephropathy**			
Number of patients included	26			25			
Recipient gender (male/female)	16/10			23/2			*P* = 0.010
Age of recipient (years)	46.5 [24; 69]			39 [18; 60]			*P* = 0.095
Donor gender (male/female)	14/11			6/19			*P* = 0.021
Age of donor (years)	54 [19; 77]			51 [19; 62]			*P* = 0.384
Donor type (cadaveric/living)	21/5			16/9			*P* = 0.180
Retransplantation (first/second/later)	23/2/1			25/-/-			
PRA max (%)	5.5 [0; 94]			3 [0; 30]			*P* = 0.177
HLA mismatch	4 [0; 5]			3 [1; 6]			*P* = 0.315
Cold ischemia (hours)	14.28 [0.45; 26.53]			17.43 [0.35; 24.68]			*P* = 0.337
Creatinine at the biopsy (μmol/L)	255.9 [98.0; 456.8]			184 [104.7; 616.2]			*P* = 0.020
Proteinuria at the biopsy (g/24 h)	3.52 [0.14; 10.38]			2.2 [0.22; 10.62]			*P* = 0.625
Dialysis vintage (years)	1.05 [0; 4.8]			1.2 [0; 7]			*P* = 0.678
Biopsy after transplantation (years)	5.96 [1.4; 20.65]			6.71 [1.01; 17.64]			*P* = 0.735
Immunosuppression prior to biopsy	Tacrolimus-based	Cyclosporin-based	Other	Tacrolimus-based	Cyclosporin-based	Other	
	21	2	3	19	4	2	*P* = 0.623

**Table 3:** Demographic and clinical characteristics of control groups.

	**Native kidney IgAN**	**Stable graft at 3 month**
Number of patients included	11	14
Recipient gender (male/female)	7	5
Age of recipient (years)	43 [25; 67]	62 [20; 72]
Donor gender (male/female)	x	10
Age of donor (years)	x	51 [22; 67]
Donor type (cadaveric/living)	x	13/1
Retransplantation (first/second/later)	x	13/1/0
PRA max (%)	x	27 [0; 84]
HLA mismatch	x	3 [2;4]
Cold ischemia (hours)	x	14 [1;19]
Dialysis vintage (years)	x	2 [0;8]
Creatinine at the biopsy (μmol/L)	116 [69;362]	96 [70;153]
Proteinuria at the biopsy (g/24 h)	1 [0;4.5]	0 [0;0.6]

*Data are expressed as median [min;max]*.

### Biopsy sample processing and cryopreservation

All biopsies (either protocol biopsies or case biopsies) were processed by standard techniques for light microscopy (LM) and immunofluorescence (IF). Briefly, samples for LM were fixed in formalin, embedded into paraffin, cut at 2–3 μm thick serial levels and stained with hematoxylin and eosin (HE), HE with elastics, periodic acid-Schiff reagent, sirius red, and Jones methenamine silver. Sections for IF were frozen, cut in a cryostat at 4–5 μm and reacted with fluorescein-tagged antibodies to C4d, IgG, IgA, IgM, C3, and light chains (kappa, lambda). All residual fresh tissue was frozen after processing and preserved at −80°C for future molecular diagnostics.

The histological evaluation of samples was performed at the time of biopsy according to relevant Banff' classification (16–19).

### RNA isolation and reverse transcription

All samples included in the study were thawed, disrupted and homogenized; the RNA was extracted using the RNeasy Micro Kit (Qiagen), according to manufacturer's instructions; the concentration of the isolated RNA was determined by ultraviolet-visible spectrophotometer (NanoDrop 2000, Thermo Scientific). The RNA isolation method routinely used in our laboratory was validated and standardized on reference samples, in order to eliminate any errors and ensure the constancy across all measurements. The RNA isolated from tissue samples was reverse-transcribed into cDNA using theSuperScript® II Reverse Transcriptase (Invitrogen).

### Quantitative RT-PCR expression analysis

Eleven genes were selected for expression analysis on the basis of their importance in the complement cascade and its regulation, and also the existence of previous findings showing their association with chronic antibody-mediated rejection and/or recurrent IgA nephropathy: *C3, C5, CD46, CD55, CD59, C4BP, CFH, CFI, CFP, CR1*, and *C1-INH* (20–22). Additionally, *PPIA* was included as a reference gene, having been previously selected from a panel of 32 genes. The selection was made using the TaqMan® Array Human Endogenous Control (Thermo Scientific), with 5 randomly selected kidney biopsy samples from kidney transplant recipients. The gene with the most stable expression (i.e., *PPIA*) was chosen as the most reliable reference gene using the NormFinder (moma.dk/normfinder-software). The gene expression profiles were determined by quantitative RT-PCR method. The samples were processed in triplicate with a predesigned TaqMan® Gene Expression Assay (Hs00163811_m1 for *C3*, Hs01004342_m1 for *C5*, Hs00611257_m1 for *CD46*, Hs00892618_m1 for *CD55*, Hs00174141_m1 for *CD59*, Hs01103672_m1 for *C4BP*, Hs00962373_m1 for *CFH*, Hs00989715_m1 for *CFI*, Hs00175252_m1 for *CFP*, Hs00559348_m1 for *CR1*, Hs00163781_m1 for *SERPING1* [*C1*-*INH*], and Hs99999904_m1 for *PPIA*) and the TaqMan® Fast Advanced Master Mix (Applied Biosystems). The qRT-PCR amplification was performed on the ABI Prism® 7900 H.T. Sequence Detection system (Applied Biosystems). Relative quantification analysis was carried out using the 96-well plates. RQ Manager 1.2 software (Applied Biosystems) was used for the automated data analysis. Based on the results of qRT-PCR, two genes—*C4BP* and *CFP*—were excluded from the statistical analysis because in more than half of the samples the expression in these genes was too low and could not be used for further analysis.

### Immunohistochemical staining

In order to observe the differences in the surface presence of MCP protein in relation to its gene expression, 5 patients with the lowest expression and 5 patients with the highest expression of *CD46* gene from the cAMR group were selected for immunohistochemical staining. Apart from MCP, C5 staining was also performed in order to determine the overall activity of complement cascade on the cell surface. Immunohistochemical detection of MCP (clone 3F1, NovusBio, Abingdon, UK) was performed on 4-μm thick sections of paraffin-embedded tissues using the Ventana Benchmark Ultra system (Tucson, AZ, USA) with ultraView Universal DAB Detection Kit. Immunohistochemical detection of complement C5 (polyclonal antibody, ThermoFisher Sci., IL, USA) was performed on 4 μm-thick paraffin sections using a two-step indirect method. After deparaffinization and rehydration antigen retrieval was performed using heat-induced epitope retrieval in buffer pH 6.0. Endogenous peroxidase was blocked by 0.3% H_2_O_2_ in 70% methanol for 30 min. Primary antibody was applied for 60 min at room temperature and detection of antibody was performed using Simple Stain MAX PO (MULTI) (Histofine, Nichirei, Japan). Finally, specimens were stained with Dako Liquid DAB+ Substrate-Chromogen System (Dako, Glostrup, Denmark) for 5 min and were counterstained with Harris's hematoxilin. All samples were correlated with the positive controls.

### Statistical analysis

Data were analyzed using GraphPad Prism 5 software (GraphPad Software Inc.) and Sigma Plot 11 (Systat software Inc.). The normality of data distribution was verified with the Kolmogorov-Smirnov test. All data sets failed to exhibit the standard normal distribution, therefore non-parametric statistical methods were used for further analysis. The comparisons of data sets were performed using the Mann-Whitney *U*-test, chi-squared test was used for comparing categorical variables, and the correlations were performed using the Spearman correlation. Patient survival among groups was compared using the Kaplan-Meier survival analysis, with the log-rank test used to determine *p*-value. To evaluate whether particular complement gene expressions could be used to discriminate between patients in whom graft failed up to 2 years from diagnostic biopsy and those with functional graft, receiver operating characteristic (ROC) curve and calculation of the area under the curve (AUC) was performed.

## Results

### Graft survival

**Figure 1:**
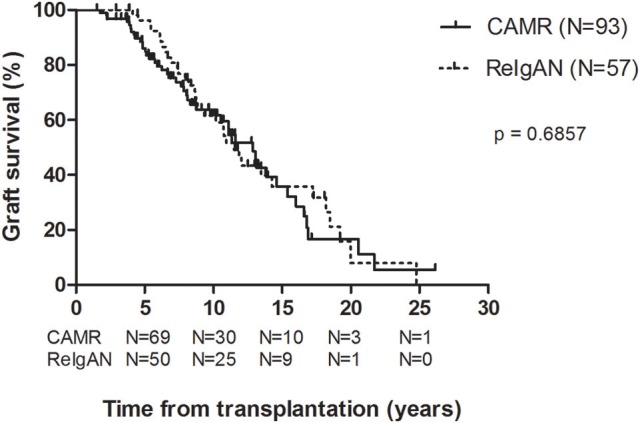
The graft survival comparison of cAMR (*N* = 93) and ReIgAN (*N* = 57) kidney allograft recipients included in the study using the Kaplan-Meier analysis; *p*-value was determined by the log-rank test.

The clinical data pertaining to the kidney allograft survival were obtained from the hospital database. Interestingly, the comparison of death-censored kidney graft survival revealed no statistically significant difference between the groups with the hazard ratio 1.097 (95% confidence interval: 0.7–1.72, *P* = 0.6857; Figure 1). The lack of significant difference suggests similarity of the impact both diagnoses have on the fate of the transplanted kidney.

### Gene expression analysis

**Figure 2:**
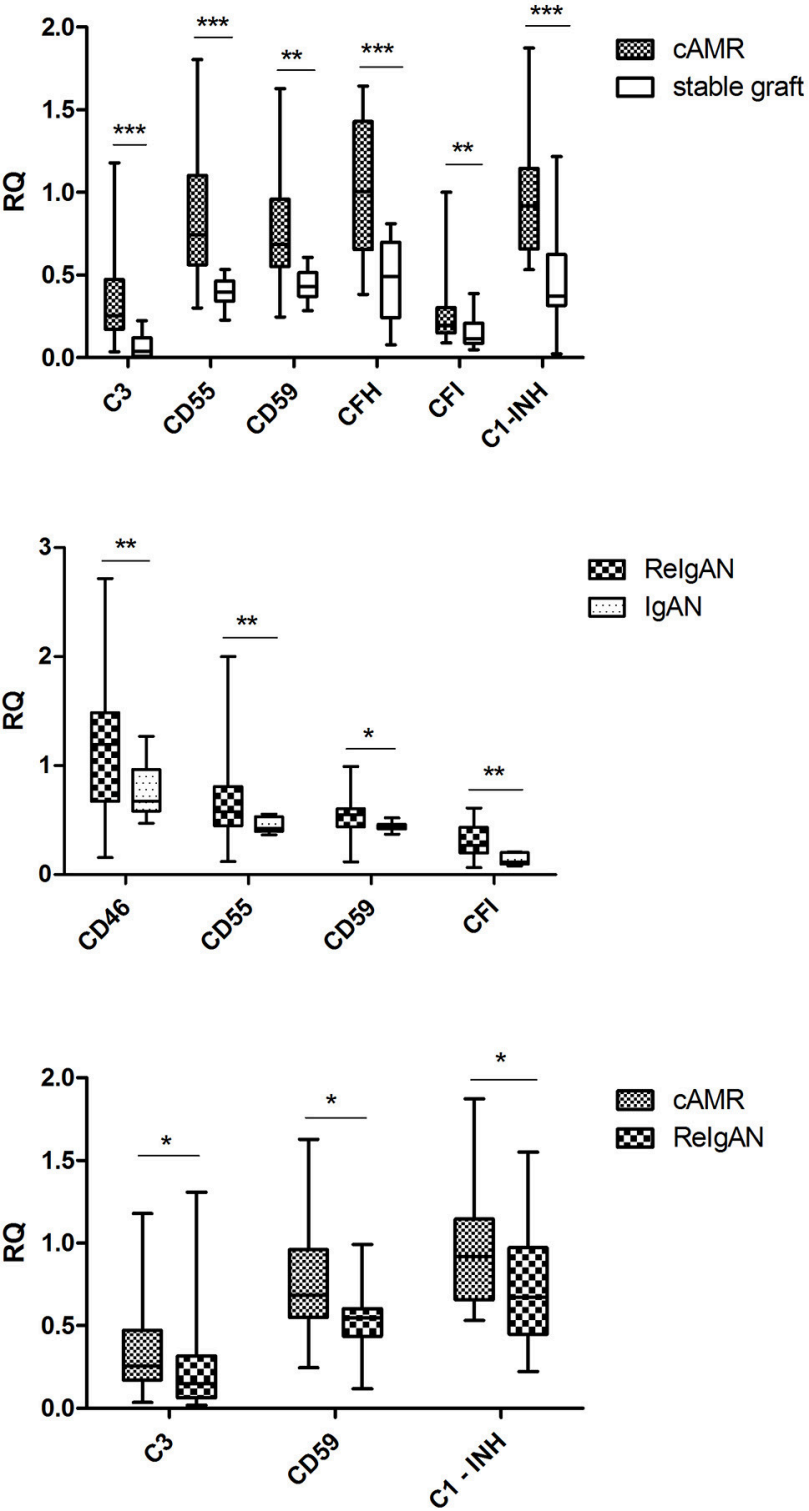
The comparison of relative quantifications in the complement genes; only the genes with statistically significant difference are included. The Mann-Whitney *U*-test was used to determine the differences. ^*^p < 0.05; ^**^p < 0.01; ^***^p < 0.001.

Interestingly, several complement gene transcripts (*C3, CD55, CD59, CFH, CFI*, and *C1-INH*) were elevated in cAMR compared to stable grafts that underlines the complement overactivity in chronic rejection (Figure 2). Moreover, higher transcripts of *CD46, CD55, CD59*, and *CFI* were observed in recurrent IgAN nephropathy than in native kidney IgA nephropathy. Intrarenal complement *C3, CD59*, and *C1-INH* mRNA transcripts were increased in the cAMR group as compared to ReIgAN, indicating the high complement activity associated with the antibody-mediated rejection (Figure 2).

**Figure 3:**
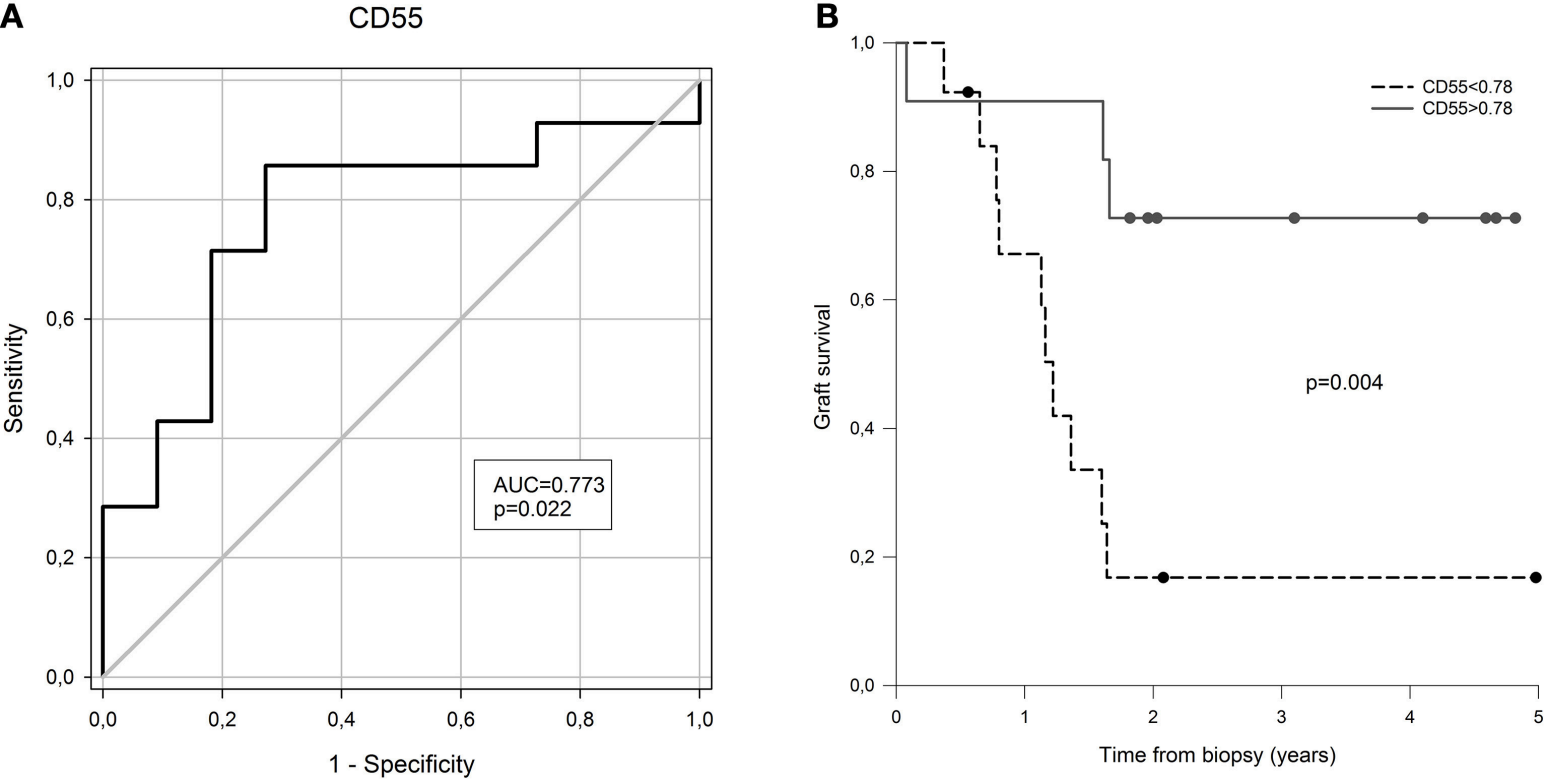
The prognostic value of *CD55* expression for graft failure in 2 years after CAMR diagnostic biopsy. **(A)** Receiver operator curve based on *CD55* gene expression at the time of biopsy. **(B)** Kaplan-Meier free of graft failure survival estimates for *CD55* gene expression above and below the cut-off point defined by ROC analysis.

Next, we evaluated possible associations of measured transcripts with rapid disease progression, defined as graft failure within 2 years from diagnostic biopsy. Interestingly, there were only borderline correlations of complement regulatory protein (*CD55, p* = 0.038) with disease progression. To find out whether the expression of *CD55* discriminates patients with graft failure at 2 years from diagnostic biopsy, we performed receiver operating characteristic curve analysis (ROC). ROC analysis confirmed lower expression of *CD55* as a risk factor for rapid progression of cAMR (Figure 3, AUC: 0.773, SE = 0.099; cut-off = 0.78 at 78.6% sensitivity and 72.7% specificity).

**Figure 4:**
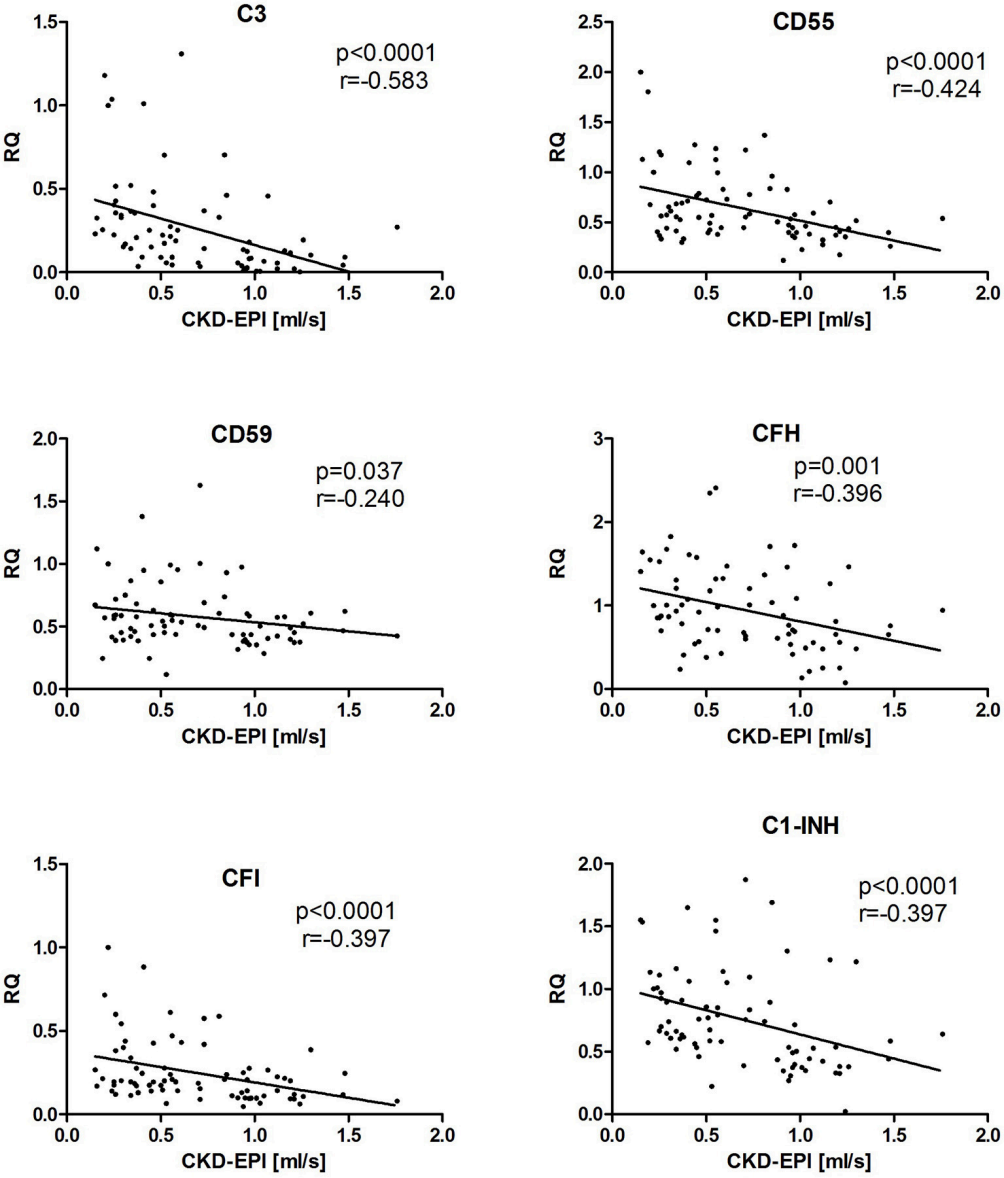
The correlation between the expression of complement genes and eGFR in kidney allograft recipients with stable function, cAMR, and ReIgAN and in IgAN of native kidney. The Spearman correlation was used to determine *p*-value and the correlation coefficient. The results are shown only for the genes where the statistical significance was found.

Finally, we evaluated possible correlation of measured intrarenal complement transcripts with a chronic kidney disease stage, defined as estimated glomerular filtration rate (eGFR). To obtain larger patient cohort for this analysis, we grouped all evaluated cohorts into a single one. There were statistically strong correlations of higher CKD stage (i.e., lower eGFR) with higher complement intrarenal transcripts, such as C3, CD59, CFH, CFI, and C1-INH (Figure 4).

### C5 and CD46 protein staining

To explain whether low or high intrarenal mRNA transcripts correlates with different protein synthesis, we performed also immunohistological substudy within cAMR group. The biopsy specimens from patients with lowest and the highest *CD46* transcripts were stained by MCP monoclonal antibody and visualized. Similarly, those biopsies were stained also with C5 antibody to prove increased complement cascade activity on the cell surface. Biopsies positively stained for MCP were negative for C5 and vice versa (Supplementary Figure 1). Neither the low mRNA transcripts samples nor the high mRNA transcripts were associated with distinct trend in MCP and C5 proteins tissue staining, respectively.

## Discussion

Complement system has been suggested to be involved in the pathogenesis of various graft pathologies, including chronic AMR and recurrent IgA nephropathy. In this retrospective study, several gene transcripts and proteins involved in complement regulation were evaluated in archive frozen tissue samples as well as in archive biopsy specimens from patients with a history of morphological diagnosis either chronic AMR or recurrent IgA nephropathy. We showed that several complement transcripts were markedly increased in those transplant pathologies compared with stable grafts or native kidney IgA nephropathy. Interestingly, there were even higher several complement gene expressions in cAMR than in recurrent IgA glomerulonephritis. Moreover, defective regulation of complement inhibitor *CD55* was shown to be associated with rapid progression of cAMR as well. Finally, advanced CKD stage correlated significantly with intrarenal expressions of several complement genes. Therefore, chronic inflammatory milieu and graft dysfunction seem to be associated with the intrarenal complement genes upregulation.

The complement overactivity in glomerular disorders is not surprising. It has been widely accepted that in proteinuric nephropathies, the increased terminal complement activity in the urine is associated with interstitial infiltrate and fibrosis (23, 24). In our study, we thus bring another evidence of this phenomenon on the molecular level.

The role of various complement system components in cAMR was shown in several studies [reviewed in (20, 21, 25)]. A recent case report of unexpected AMR developing in a patient with aHUS with identified mutations in *CFH* and *CD46* genes suggests a possible role of complement regulators in the antibody-mediated rejection as well (26). Our observation of the link between the defective *CD55* expression and the rapid cAMR progression indicates possible influence of complement decay-accelerating factor that indirectly blocks the formation of the membrane attack complex on the chronic antibody-mediated rejection outcome.

Interestingly, very recently, expression pattern of *CD46* and *CD55*, both complement protein regulators were studied in the peripheral blood in patients with progressive IgAN nephropathy (27). The study showed that defective peripheral *CD46* gene expression did not correlate with eGFR at sampling but with a faster annual loss of GFR. Moreover, the prognostic effect of both peripheral transcripts on IgA progression was very poor with ROC 0.51 and 0.61, respectively. However, expression patterns improve prognostic effect of clinical parameters. Similarly, in our study there were no associations of eGFR with intrarenal *CD46* complement transcripts. In our study, we described association of several intrarenal gene transcripts with CKD stage, including *CD55*. Clearly, fibrosis *per se* was shown to be associated with increased complement activity; similarly, the glomerular disease activity is known to be associated with increased renal complement as well [reviewed in (24)]. This is not surprising as complement is involved in triggering both innate and adaptive immune system. This better fits to our observation; CKD stage perfectly correlated with intrarenal complement regulation, however there were differences between cAMR and recurrent IgA nephropathy. Moreover, both transplant pathologies with already established graft dysfunction had higher expression of complement genes than patients with stable grafts and normal histology or in patients with native kidney IgA nephropathy at histological diagnosis.

The comparison of complement genes expression in the allograft tissue between cAMR and ReIgAN cohorts revealed significant differences. The transcripts of several components of complement cascade, namely C3, CD59, and C1-Inh, showed higher levels of expression in cAMR patients. C3 is the integral part of all complement activation cascades and the increased amount of its transcripts could well be explained as a sign of increased complement system activity. Its subsequent cleavage to C3a and C3b fragments promotes the complement cascade and the C3a fragment functions as a significant anaphylatoxin, supporting the inflammatory processes (28–30). Recently, Mahakur et al. discovered a significant difference in C3 expression in patients with antibody-mediated rejection, as compared to stable allograft results (31). In contrast, recent study confirmed earlier results suggesting that the decrease of C3 levels in serum is a potential predictive factor of poor renal prognosis in IgA nephropathy patients (32).

Similarly, C1-inhibitor has been studied in relation to cAMR, due to its ability to effectively block both classical and lectin activation pathways. The classical activation pathway is considered dominant in cAMR, therefore the increased expression of C1-Inh in the kidney allograft tissue might be the sign of increased complement system activity. Since the ability of HLA antibodies to bind C1q complement fraction, and subsequently activate the classical complement pathway, can significantly affect the kidney allograft outcome, the inhibition of C1 complex components may provide another therapeutic target (33). Tillou et al. used a non-human primate model to examine the potential of C1-Inh to suppress acute antibody-mediated rejection (34). More recently, the application of C1-Inh was studied in kidney transplant recipients, revealing possible positive influence on C1q+ HLA antibody levels and the occurrence of antibody-mediated rejection (14), which was again confirmed in later study (35).

While C1-Inh is able to block the complement activation, CD59 functions as a regulator of the terminal cascade and interferes with the membrane attack complex formation (36). The expression of CD59 was suggested previously to be a protection mechanism of donor organs against the complement-mediated injury (37), its increased expression in cAMR patients might therefore point to the general increase in complement system activity.

In our study, we found no correlation of the immunohistochemical staining results with the gene expression analysis results. The lack of distinct trend could signify that complement regulator genes activation and transcription might not necessarily lead to the generation and expression of finalized protein. Furthermore, MCP acts as a cofactor for complement Factor I and is unable to interrupt the complement cascade on its own. Therefore, higher expression of MCP without the markedly increased expression of CFI protein might not influence the complement activity on the cell surface.

Interestingly, the results of our study suggest similar kidney graft survival in recurrent IgA nephropathy and cAMR. The recurrent IgA nephropathy was formerly presumed to affect the long-term kidney graft survival only in small and relatively insignificant way, compared to other post-transplant pathologies (23). Recent studies with longer follow-up showed, however, that the IgA nephropathy recurrence can indeed be considered as an independent predictor of worsened graft outcome (5). This surprising observation needs to be further validated. Clearly, our study had retrospective design, however long-term patient's follow-up in a single center minimized potential bias.

There are also several weaknesses of our study which could not be addressed. Aside of retrospective nature, anti-HLA antibodies could not be determined in cAMR cohort as many biopsies were performed before the technique was available and sera were not stored for such a long time. Therefore, the diagnosis of cAMR was strictly histological. Similarly, we cannot rule out the influence of DSA on the graft outcome also in patients with recurrent IgA nephropathy. Moreover, similar long-term kidney graft outcome in both studied cohorts may suggest undetected humoral alloimmune response at the time when biopsy was performed or subsequent humoral response later after recurrent disease diagnosis.

In summary, our study shows that the long-term renal allograft survival in patients with histologically diagnosed chronic antibody-mediated rejection and recurrent IgA nephropathy is similar. In both processes, the increased complement cascade transcripts were present, however cAMR was associated with higher levels of their expression levels. The intrarenal complement system transcripts are upregulated in progressively deteriorated kidney allografts and thus may represent potential target for innovative therapeutics.

## Author contributions

MC and MK performed the research and wrote the manuscript. PH, AL, PM, LS, and EH performed the research. OV supervised the project and wrote the manuscript.

### Conflict of interest statement

OV received speaker fees from Alexion, Astellas, Chiesi, Novartis, and Sanofi. The remaining authors declare that the research was conducted in the absence of any commercial or financial relationships that could be construed as a potential conflict of interest. The reviewer FF and the handling editor declared their shared affiliation.

## Supplementary material

Supplementary Figure 1 The results of immunohistochemical staining; CD46-stained samples are presented in the top rows, C5-stained samples in the bottom rows. **(A)** 5 cAMR patients with the lowest CD46 transcript levels; **(B)** 5 cAMR patients with the highest CD46 transcript levels.

## Abbreviations

cAMR: chronic antibody-mediated rejection
C1-Inh: C1-inhibitor
eGFR: estimated glomerular filtration rate
HE: hematoxylin and eosin
HLA: human leukocyte antigen
IF: immunofluorescence
LM: light microscopy
MCP: Membrane Cofactor Protein
PRA: panel reactive antibodies
ReIgAN: recurrent IgA nephropathy
RT-PCR: real-time quantitative polymerase chain reaction.

## References

[1] Einecke G Sis B Reeve J Mengel M Campbell PM Hidalgo LG. Antibody-mediated microcirculation injury is the major cause of late kidney transplant failure. Am J Transplant. (2009) 9:2520–31. 10.1111/j.1600-6143.2009.02799.x 19843030

[2] Gaston RS Cecka JM Kasiske BL Fieberg AM Leduc R Cosio FC. Evidence for antibody-mediated injury as a major determinant of late kidney allograft failure. Transplantation (2010) 90:68–74. 10.1097/TP.0b013e3181e065de 20463643

[3] Haas M. An updated Banff schema for diagnosis of antibody-mediated rejection in renal allografts. Curr Opin Organ Transplant. (2014) 19:315–22. 10.1097/MOT.0000000000000072 24811440

[4] Chand S Atkinson D Collins C Briggs D Ball S Sharif A. The spectrum of renal allograft failure. PLoS ONE (2016) 11:e0162278. 10.1371/journal.pone.0162278 27649571 PMC5029903

[5] Moroni G Longhi S Quaglini S Gallelli B Banfi G Montagnino G. The long-term outcome of renal transplantation of IgA nephropathy and the impact of recurrence on graft survival. Nephrol Dial Transplant. (2013) 28:1305–14. 10.1093/ndt/gfs472 23229925

[6] Maixnerova D Jancova E Skibova J Rysava R Rychlik I Viklicky O. Nationwide biopsy survey of renal diseases in the Czech Republic during the years 1994-2011. J Nephrol. (2015) 28:39–49. 10.1007/s40620-014-0090-z 24756969

[7] Rauterberg EW Lieberknecht HM Wingen AM Ritz E. Complement membrane attack (MAC) in idiopathic IgA-glomerulonephritis. Kidney Int. (1987) 31:820–9. 10.1038/ki.1987.72 3573542

[8] Brodsky SV Nadasdy GM Pelletier R Satoskar A Birmingham DJ Hadley GA Expression of the decay-accelerating factor (CD55) in renal transplants–a possible prediction marker of allograft survival. Transplantation (2009) 88:457–64. 10.1097/TP.0b013e3181b0517d 19696627

[9] Onda K Ohsawa I Ohi H Tamano M Mano S Wakabayashi M. Excretion of complement proteins and its activation marker C5b-9 in IgA nephropathy in relation to renal function. BMC Nephrol. (2011) 12:64. 10.1186/1471-2369-12-64 22111871 PMC3283454

[10] Park MS Kim SK Lee TW Lee SH Moon JY Ihm CG. A promoter polymorphism in the CD46 complement regulatory protein gene is associated with acute renal allograft rejection. Transplant Proc. (2016) 48:809–12. 10.1016/j.transproceed.2015.12.126 27234742

[11] Xie J Kiryluk K Li Y Mladkova N Zhu L Hou P. Fine mapping implicates a deletion of CFHR1 and CFHR3 in protection from IgA nephropathy in Han Chinese. J Am Soc Nephrol. (2016) 27:3187–94. 10.1681/ASN.2015111210 26940089 PMC5042673

[12] Stegall MD Diwan T Raghavaiah S Cornell LD Burns J Dean PG. Terminal complement inhibition decreases antibody-mediated rejection in sensitized renal transplant recipients. Am J Transplant. (2011) 11:2405–13. 10.1111/j.1600-6143.2011.03757.x 21942930

[13] Tran D Boucher A Collette S Payette A Royal V Senécal L. Eculizumab for the treatment of severe antibody-mediated rejection: a case report and review of the literature. Case Rep Transplant. (2016) 2016:9874261. 10.1155/2016/9874261 27478676 PMC4958444

[14] Vo AA Zeevi A Choi J Cisneros K Toyoda M Kahwaji J. A phase I/II placebo-controlled trial of C1-inhibitor for prevention of antibody-mediated rejection in HLA sensitized patients. Transplantation (2015) 99:299–308. 10.1097/TP.0000000000000592 25606785

[15] Wahrmann M Mühlbacher J Marinova L Regele H Huttary N Eskandary F. Effect of the anti-C1s humanized antibody TNT009 and its parental mouse variant TNT003 on HLA antibody-induced complement activation-a preclinical *in vitro* study. Am J Transplant. (2017) 17:2300–11. 10.1111/ajt.14256 28251805 PMC5600102

[16] Solez K Colvin RB Racusen LC Haas M Sis B Mengel M. Banff 07 classification of renal allograft pathology: updates and future directions. Am J Transplant. (2008) 8:753–60. 10.1111/j.1600-6143.2008.02159.x 18294345

[17] Sis B Mengel M Haas M Colvin RB Halloran PF Racusen LC. Banff '09 meeting report: antibody mediated graft deterioration and implementation of Banff working groups. Am J Transplant. (2010) 10:464–71. 10.1111/j.1600-6143.2009.02987.x 20121738

[18] Mengel M Sis B Haas M Colvin RB Halloran PF Racusen LC. Banff 2011 Meeting report: new concepts in antibody-mediated rejection. Am J Transplant. (2012) 12:563–70. 10.1111/j.1600-6143.2011.03926.x 22300494 PMC3728651

[19] Haas M Sis B Racusen LC Solez K Glotz D Colvin RB. Banff 2013 meeting report: inclusion of c4d-negative antibody-mediated rejection and antibody-associated arterial lesions. Am J Transplant. (2014) 14:272–83. 10.1111/ajt.12590 24472190

[20] Farrar CA Sacks SH. Mechanisms of rejection: role of complement. Curr Opin Organ Transplant. (2014) 19:8–13. 10.1097/MOT.0000000000000037 24316762

[21] Sheen JH Heeger PS. Effects of complement activation on allograft injury. Curr Opin Organ Transplant. (2015) 20:468–75. 10.1097/MOT.0000000000000216 26132735 PMC4510836

[22] Daha MR van Kooten C. Role of complement in IgA nephropathy. J Nephrol. (2016) 29:1–4. 10.1007/s40620-015-0245-6 26567162 PMC4733139

[23] Mosolits S Magyarlaki T Nagy J. Membrane attack complex and membrane cofactor protein are related to tubulointerstitial inflammation in various human glomerulopathies. Nephron (1997) 75:179–87. 10.1159/000189529 9041539

[24] Fearn A Sheerin NS. Complement activation in progressive renal disease. World J Nephrol. (2015) 4:31–40. 10.5527/wjn.v4.i1.31 25664245 PMC4317626

[25] Cernoch M Viklicky O. Complement in kidney transplantation. Front Med. (2017) 4:66. 10.3389/fmed.2017.00066 28611987 PMC5447724

[26] Stevenson S Mallett A Oliver K Hyland V Hawley C Malmanche T. Atypical HUS associated with severe, unexpected antibody-mediated rejection post kidney transplant. Nephrology (2014) 19(Suppl. 1):22–6. 10.1111/nep.12195 24460647

[27] Coppo R Peruzzi L Loiacono E Bergallo M Krutova A Russo ML. Defective gene expression of the membrane complement inhibitor CD46 in patients with progressive immunoglobulin A nephropathy. Nephrol Dial Transplant. (2018). [Epub ahead of print]. 10.1093/ndt/gfy064 29635535

[28] Ehrengruber MU Geiser T Deranleau DA. Activation of human neutrophils by C3a and C5A. Comparison of the effects on shape changes, chemotaxis, secretion, and respiratory burst. FEBS Lett. (1994) 346:181–4. 10.1016/0014-5793(94)00463-3 8013630

[29] Fischer WH Jagels MA Hugli TE. Regulation of IL-6 synthesis in human peripheral blood mononuclear cells by C3a and C3a(desArg). J Immunol. (1999) 162:453–9. 9886419

[30] Klos A Tenner AJ Johswich KO Ager RR Reis ES Köhl J. The role of the anaphylatoxins in health and disease. Mol Immunol. (2009) 46:2753–66. 10.1016/j.molimm.2009.04.027 19477527 PMC2725201

[31] Mahakur S Saikia B Minz M Minz RW Nada R Anand S. Allo-specific immune response profiles indicative of acute rejection in kidney allografts using an *in vitro* lymphocyte culture-based model. Clin Exp Nephrol. (2018) 22:465–73. 10.1007/s10157-017-1469-7 28849286

[32] Pan M Zhang J Li Z Jin L Zheng Y Zhou Z. Increased C4 and decreased C3 levels are associated with a poor prognosis in patients with immunoglobulin A nephropathy: a retrospective study. BMC Nephrol. (2017) 18:231. 10.1186/s12882-017-0658-7 28697742 PMC5505039

[33] Loupy A Lefaucheur C Vernerey D Prugger C Duong van Huyen JP Mooney N. Complement-binding anti-HLA antibodies and kidney-allograft survival. N Engl J Med. (2013) 369:1215–26. 10.1056/NEJMoa1302506 24066742

[34] Tillou X Poirier N Le Bas-Bernardet S Hervouet J Minault D Renaudin K. Recombinant human C1-inhibitor prevents acute antibody-mediated rejection in alloimmunized baboons. Kidney Int. (2010) 78:152–9. 10.1038/ki.2010.75 20336054

[35] Viglietti D Gosset C Loupy A Deville L Verine J Zeevi A. C1 inhibitor in acute antibody-mediated rejection nonresponsive to conventional therapy in kidney transplant recipients: a pilot study. Am J Transplant. (2016) 16:1596–603. 10.1111/ajt.13663 26693703

[36] Farkas I Baranyi L Ishikawa Y Okada N Bohata C Budai D CD59 blocks not only the insertion of C9 into MAC but inhibits ion channel formation by homologous C5b-8 as well as C5b-9. J Physiol. (2002) 539(Pt 2):537–45. 10.1113/jphysiol.2001.013381 11882685 PMC2290142

[37] Griesemer AD Okumi M Shimizu A Moran S Ishikawa Y Iorio J. Upregulation of CD59: potential mechanism of accommodation in a large animal model. Transplantation (2009) 87:1308–17. 10.1097/TP.0b013e3181a19afc 19424030 PMC2772119

